# Phenotypic Characterization of HIV-Specific CD8^+^ T Cells during Early and Chronic Infant HIV-1 Infection

**DOI:** 10.1371/journal.pone.0020375

**Published:** 2011-05-31

**Authors:** Jennifer A. Slyker, Grace C. John-Stewart, Tao Dong, Barbara Lohman-Payne, Marie Reilly, Ann Atzberger, Stephen Taylor, Elizabeth Maleche-Obimbo, Dorothy Mbori-Ngacha, Sarah L. Rowland-Jones

**Affiliations:** 1 MRC Human Immunology Unit, Oxford University, Oxford, United Kingdom; 2 Department of Global Health, University of Washington, Seattle, Washington, United States of America; 3 Department of Medicine, University of Washington, Seattle, Washington, United States of America; 4 Department of Epidemiology, University of Washington, Seattle, Washington, United States of America; 5 Department of Paediatrics, University of Nairobi, Nairobi, Kenya; 6 Medical Epidemiology & Biostatistics, Karolinska Institutet, Stockholm, Sweden; 7 Molecular Haematology Unit, Weatherall Institute of Molecular Medicine, Oxford University, Oxford, United Kingdom; 8 Computational Biology Research Group, Weatherall Institute of Molecular Medicine, Oxford University, Oxford, United Kingdom; Massachusetts General Hospital, United States of America

## Abstract

Although CD8^+^ T cells play an important role in the containment of adult HIV-1 replication, their role in infant HIV-1 infection is not as well understood. Impaired HIV-specific CD8^+^ T cell responses may underlie the persistently high viral loads observed in infants. We examined the frequency and phenotype of infant HIV-specific CD8^+^ T cells in 7 HIV-infected antiretroviral therapy-naïve infants during the first 2 years of life, using class I HLA tetramers and IFN-γ-ELISPOT. The frequency (0.088–3.9% of CD3^+^CD8^+^ cells) and phenotype (CD27^+^CD28^−^, CD45RA^+/−^, CD57^+/−^, HLA-DR^+^, CD95^+^) of infant HIV-specific CD8^+^ T cells were similar to reports in adults undergoing early infection. Unlike adults, at 23–24 months post-infection a high frequency of HIV-specific CD8^+^ T cells expressed HLA-DR (mean 80%, range 68–85%) and CD95 (mean 88%, range 79–96%), suggesting sustained activation and vulnerability to apoptosis. Despite comparable expansion of HIV-specific CD8^+^ T cells of a similar phenotype to adults during early infection, infant T cells failed to contain HIV-1 replication, and remained persistently activated and vulnerable to apoptosis during chronic infection.

## Introduction

The natural history of human immunodeficiency virus type-1 (HIV-1) infection in infants differs markedly from adults. In adults, plasma viral load peaks during acute infection and is subsequently reduced to a >1 log lower level set-point over the next 6–8 weeks [1]. This viral load set-point may be maintained for years, sometimes at levels below detection. In contrast, infants experience very high plasma viral loads (often more than 1 million copies/ml) and lack the characteristic decline to set-point observed in adults [2], [3], [4], [5], [6]. Infant CD4^+^ T cells are rapidly depleted during the first year of life and there is much morbidity due to opportunistic infection. In the absence of antiretroviral therapy (ART), 2-year mortality rates as high as 52% have been reported in African cohorts [7], [8], [9], [10].

Adult studies of HIV-1 infection and SIV models have demonstrated the importance of HIV-specific CD8^+^ T cells in limiting viral replication [11], [12], [13], [14]. The rapid HIV-1 disease progression experienced by infants may be a consequence of suboptimal T cell responses during early life. Studies conducted in perinatally infected infants report less frequent detection of CD8^+^ T cell responses, and responses of a smaller magnitude than are typically observed in HIV-infected adults [15], [16]. There are conflicting findings regarding the association between CD8^+^ T cell responses and HIV-1 viral load or disease progression in infants and children; with some studies showing a positive, negative, or absence of correlation [16], [17], [18], [19], [20]. The detection of escape mutations suggests some infant CD8^+^ T cells exert sufficient selection pressure to drive viral evolution [21], [22]. In 85 vertically infected infants examined longitudinally during the first year of life, we observed an increase in the magnitude of HIV-specific IFN-γ responses over time [17]. We also found infants who acquired HIV-1 after 1 month of age were able to generate IFN-γ responses more rapidly than infants with *in utero* or peripartum infection [23]. Together these data suggest that the capacity of the infant cellular immune system to generate HIV-specific IFN-γ responses increases rapidly in the first months of life.

Understanding the specific mechanisms by which infant T cells fail to contain HIV-1 is important to the design of vaccines appropriate for use in infants. In addition, defects in infant immune responses that explain poor viral control may contribute more broadly to our understanding of HIV-1 immune pathogenesis. In this study, we longitudinally describe the frequency and phenotype of HIV-specific CD8^+^ T cells during primary and chronic HIV-1 infection in a group of Kenyan infants.

## Results

### Characteristics of selected participants

The results of IFN-γ-ELISPOT assays performed in the previously characterized cohort study [17] were used to select infants with high-level responses for more detailed phenotypic studies using class I HLA tetramers. A total of 85 children became infected during the course of the study, 72 acquired HIV-1 before the first month of life, and 61 of these had ELISPOT data. Of these 61 infants with early HIV-1 acquisition, 26 (43%) had ELISPOT responses greater than 400 HIVSFU/million PBMC. Of these, 7 infants had responses directed at HIV-1 epitopes for which we were able to construct tetramers and had sufficient cells cryopreserved for flow cytometry studies. Table 1 shows the virologic and immunologic characteristics for the selected infants. The median peak response to these peptides was 993 HIVSFU/10^6^ PBMC (Table 1, range 450–2040). HIV-1 RNA viral loads were high, the mean peak was 6.7 log_10_ (SD ±0.65) and the mean set-point was 6.2 log_10_ copies/ml (SD ±0.53). The level of viral replication in these seven infants was representative of the overall cohort from which the children were selected (mean peak and set-point viral loads of whole infected cohort 6.8, SD ±0.74, p = 0.7; 6.2 log_10_ SD ±0.66 copies/ml, p = 1.0). At 6 months of age, the selected infants' median CD4 percentage was 26% (IQR = 21–29%); similar to that of the overall infected cohort (21%, IQR  = 12–28%, p = 0.2). Six of the 7 infants had CD4 percentages meeting WHO criteria [24] for advanced (3 infants) or severe (3 infants) immunosuppression and two did not survive the 2-year follow-up period. The HLA-A29 allele, which was associated with a trend for increased risk of early HIV-1 acquisition in the overall cohort [25], was present in 4 of the children studied here. HLA-A29 is common in Kenya and 13% (8/61) of children with early HIV-1 infection in the cohort had this allele. The A29-GP120 epitope was among the most immunodominant epitopes targeted in the cohort; 5 of 8 (63%) HLA-A29^+^ children had positive ELISPOT responses to A29-GP120 during the first year of life [17].

**Table 1:** Infant characteristics.

Infant	HIV infection	Log HIV peak	Log HIV set-point	Month 6 CD4%	MonthsFollow-up	Class I HLA type	HLA restriction and immunodominant epitope	Maximum IFN-γ-ELISPOT response in year 1 (per 10^6^ PBMC)
B1-045	*in utero*	6.3	5.2	35	24	A1, A3, B15, B57, Cw4, Cw7	A*03 Nef-QVPLRPMTYK	1170 HIVSFU
B1-093	*in utero*	6.7	6.4	21	24	A29, A74, B14, B1503, Cw2	A*29 GP120-FNCGGEFFY	993 HIVSFU
B1-159	peripartum	6.9	5.8	26	24	A29, A30, B4501, Cw6	A*29 GP120-FNCGGEFFY	723 HIVSFU
B1-170	*in utero*	5.9	5.2	21	18^b^	A30, A3402, B42, B57, Cw7, Cw17	B*57 P24-KAFSPEVIPMF	1433 HIVSFU
B1-291	peripartum	7.2	6.8	14	15^b^	A29, A74, B42, B15, Cw2, Cw17	A*29 GP120-FNCGGEFFY	685 HIVSFU
B1-334	peripartum	6.5	6.2	26	9^b^	A29, A26, B13, B15, Cw2, Cw6	A*29 GP120-FNCGGEFFY	2040 HIVSFU
B1-454	peripartum	7.6	7.1	29	24	A2, A31, B8, B15, Cw7, Cw8	B*08 Nef-FLKEKGGL	450 HIVSFU

a B1-045 did not have CD4 data at 6 months, data from 9 months are shown.

b B1-170 was declared lost to follow-up, B1-291 and 334 died at 17 and 11 months of age, respectively.

### Frequencies of HIV-specific CD8^+^ T cells

**Figure 1:**
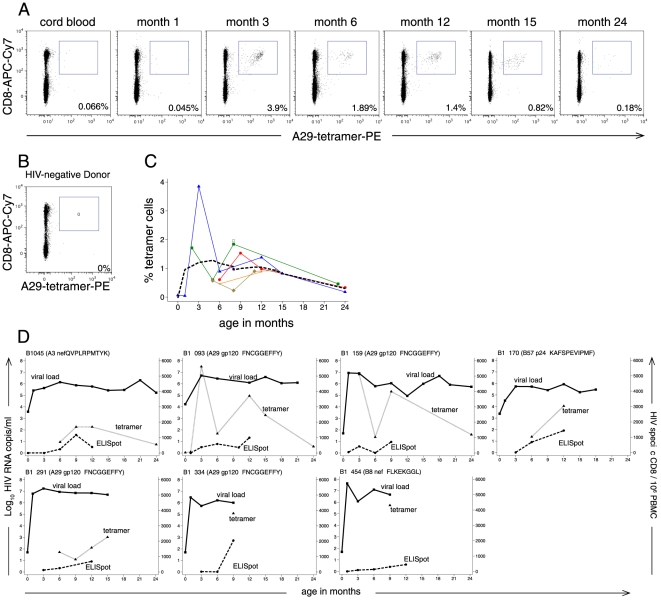
Tetramer-staining reveals the expansion of HIV-1 specific CD8 T cells during acute infant HIV-1 infection. A) Representative HLA-A29 GP120 tetramer staining from B1-093, who acquired HIV-1 *in utero*. Plots show cells gated on CD3+ lymphocytes. Frequencies of tetramer-positive cells are provided as a percent of CD3^+^CD8^+^ gated cells. B) Tetramer staining from a negative control donor. C) Frequency of tetramer-positive cells, expressed as a percentage of the CD3^+^CD8^+^ subset. Colored lines and markers show individual infants, bold dashed line shows a median spline. D) HIV-1 RNA viral load (solid line with squares, left y-axis), ELISPOT HIVSFU/million PBMC (dashed line with circles, right y-axis), and Tetramer+/10^6^ PBMC (HIV-specific CD8+ T cells, dotted line with triangles, right y-axis) are shown for each of the 7 infants. Note, ELISPOT assays were conducted during live cohort follow-up for 1 year, viral load was measured at each study visit over 2 years of follow-up, tetramer staining was performed on all available frozen specimens. Missing values at visits indicate missed visit or specimen not available for assay.

Seven infants provided a median of 4 cryopreserved specimens (range 1–7 specimens) for tetramer staining. Tetramer staining detected HIV-specific CD8^+^ T cells in all 7 infants. Figure 1A shows an example of tetramer staining in subject B1-093 who was diagnosed with HIV-1 infection at birth. Though this infant had acquired HIV-1 *in utero*, no responses to the HLA-A29 GP120 epitope were detected by ELISPOT until 3 months of age, at which time 3.9% of CD3^+^CD8^+^ T cells were tetramer-positive. There was a decline in cells detected by tetramer in infant B1-093 after 3 months of age, though a discrete population of A29-GP120 specific cells remained discernable at 24 months. The median peak frequency of tetramer staining in the group of infants was 1.5% of CD3^+^CD8^+^ cells (range 0.088–3.9%) and declined thereafter (Figure 1C).

Figure 1D shows tetramer staining, ELISPOT, and HIV-1 viral load data for each infant. In order to compare HIV-specific CD8 T cells identified by tetramer and ELISPOT, we show the number of tetramer-positive cells/10^6^ PBMC. Tetramer staining consistently detected more HIV-epitope-specific CD8^+^ T cells than ELISPOT, in agreement with previous reports that only a subset of HIV-specific CD8^+^ T cells secrete IFN-γ [26], [27]. Although substantial numbers of tetramer-positive and IFN-γ-secreting HIV-specific CD8^+^ T cells were detected during infant HIV-1 infection, we observed minimal decline in HIV-1 viral loads during study follow-up; viral loads remained above 5 log_10_ copies/ml in all 7 infants and did not appear to follow a pattern similar to either tetramer-positive or IFN-γ-secreting cell frequencies.

### Phenotypic characterization of infant HIV-specific CD8^+^ T cells

**Figure 2:**
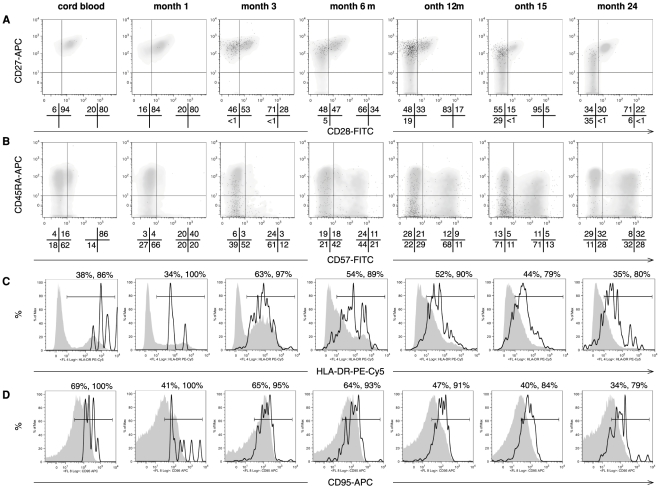
Differentiation of infant CD8 T cells during early and chronic infant HIV-1 infection. Dot plots and histograms show representative phenotyping during the first two years of life in subject B1-093, who was HIV-1 RNA positive at birth. Dot plots show A) CD27 and CD28 and B) CD45RA and CD57 staining. HIV-specific CD8^+^ T cells (gated on tetramer^+^/CD3^+^CD8^+^) are shown as black dot plots overlaid on the overall CD8^+^ subset (gated on CD3^+^CD8^+^), which are shown as density plots in gray. Quadrant statistics are shown below each dot plot, for the overall CD8^+^ (left diagram) and HIV-specific CD8^+^ T cell subsets (right diagram). Histograms show C) HLA-DR and D) CD95 staining, the overall CD8^+^ subset is shown by the solid gray histogram, and the HIV-specific CD8^+^ T cell subset is overlaid in black outline as an unshaded histogram. Histogram gate percentages are shown above the plots for the overall CD8^+^, and HIV-specific CD8^+^ T cell subsets, respectively.

Analysis of the overall CD8^+^ and HIV-specific CD8^+^ T cell subsets demonstrated a phenotype similar to that observed in adults undergoing acute HIV-1 infection. Figure 2 shows longitudinal CD8^+^ T cell phenotype of a representative infant infected with HIV-1 *in utero*. Though the overall CD8^+^ T cell subset was a homogenous mixture of CD27^+^CD28^+^ (early), CD27^+^CD28^−^ (intermediate) and CD27^−^CD28^−^ (late) differentiated cells, HIV-specific CD8^+^ T cells were predominantly CD27^+^CD28^−^ (Figure 2A). A subset of CD3^+^CD8^+^ expressed both CD45RA and CD57, suggesting the generation of terminally differentiated effector cells (Figure 2B). Consistent with their role as effector cells, CD45RA^+^CD57^+^ cells expressed high levels of HLA-DR, indicative of activation (data not shown). Both the overall CD8^+^ and HIV-specific CD8^+^ T cell subsets contained high frequencies of HLA-DR^+^ (Figure 2C) and CD95^+^ cells (Figure 2D).

### Longitudinal changes in T cell phenotype during infant HIV-1 infection

**Figure 3:**
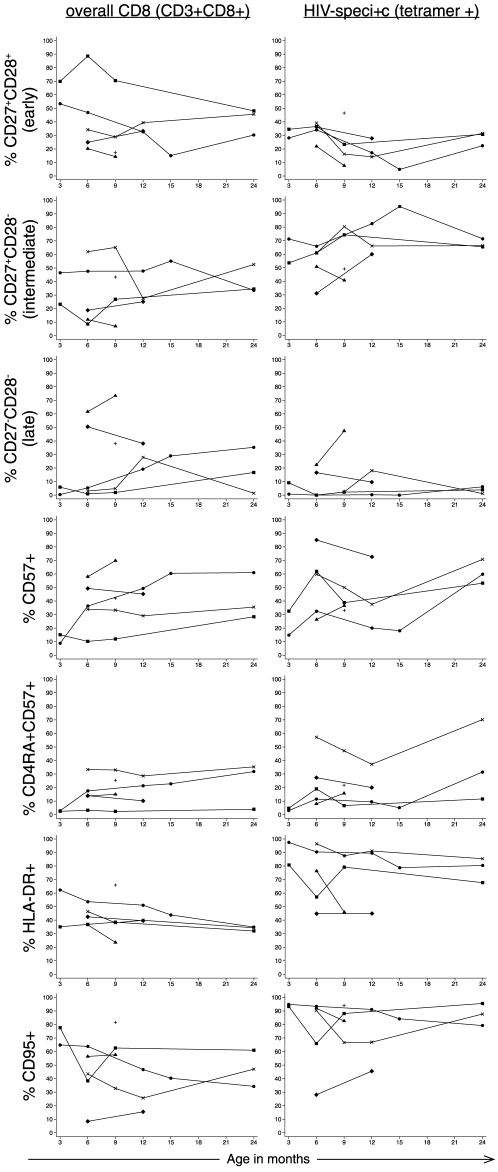
Changes in the overall and HIV-specific CD8^**+**^ T cell subsets during the first two years of infection. The frequency of HIV-specific CD8^+^ T cells and overall CD8^+^ T cells expressing each marker or combination of markers are shown for the first two years of life. All infants were infected by 1 month of age. Connected gray lines show individual infants with more than 1 measurement of phenotype (N = 5), one infant had only one time point analyzed for phenotype (B1-334, + symbol).

Figure 3 shows longitudinal data from early through chronic infection in the overall CD8^+^ and HIV-specific CD8^+^ T cell subsets. Since the study specimens were collected at a variety of time-points, we created time intervals of 3–6 months, 7–12 months and 23–24 months post HIV-1 infection to enable quantitative comparisons of overall CD8^+^ and HIV-specific CD8^+^ T cell subsets and the description of changes over time (Table 2). HIV-specific CD8^+^ T cells were highly polarized toward an intermediate, activated phenotype compared to the overall CD8^+^ T cell subset. The frequency of CD27^+^CD28^−^ cells was higher in the HIV-specific CD8^+^ T cell subset (mean 73%, range 41–83) compared to the overall CD8^+^ T cell subset (27%, range 7.0–48) during the 7–12 month interval (p = 0.018). HLA-DR^+^ cells were detected at high levels in the HIV-specific CD8^+^ T cell subset compared to the overall CD8^+^ subset at 3–6 months (81%, range 45–97 versus 43%, range 35–62, respectively; p = 0.017), 7–12 months (79%, range 45–90 versus 39% range 24–51, respectively; p = 0.005), and 23–24 months post HIV-1 infection (80%, range 68–85 versus 34%, range 32–35%, respectively; p = 0.0032). Similarly, CD95^+^ cells were detected at a higher frequency in the HIV-specific CD8^+^ T cell subset compared to the overall CD8^+^ subset at 3–6 months (92%, range 28–95 versus 56%, range 8.4–78, respectively; p = 0.027), 7–12 months (83%, range 46–91 versus 47%, range 16–63, respectively; p = 0.010), and 23–24 months (88%, range 79–96 versus 47%, range 34–61, respectively; p = 0.030) post HIV-1 infection.

**Table 2:** Comparison of overall CD8 and HIV-specific phenotype over the first 2 years of HIV-1 infection.

	% (range) cells with phenotype in each time interval			
	3–6 months	7–12 months	23–24 months	*p* *
Number of infants	5	5	3	
CD27^+^CD28^+^ (early)				
Overall CD8	34 (20–70)	33 (14–71)	46 (30–48)	*0.49*
HIV-specific	35 (22–39)	17 (7.7–28)	31 (22–32)	*0.036*
*p* **	*0.53*	*0.25*	*0.012*	
CD27^+^CD28^−^ (intermediate)				
Overall CD8	23 (12–62)	27 (7.0–48)	35 (34–53)	*0.90*
HIV-specific	54 (31–71)	73 (41–83)	66 (65–71)	*0.23*
*p* **	*0.059*	*0.018*	*0.076*	
CD27-CD28^−^ (late)				
Overall CD8	5.9 (0.38–62)	19 (1.9–73)	17 (1.4–35)	*0.41*
HIV-specific	9.1 (0–22)	9.6 (0.29–47)	3.9 (1.1–6.1)	*0.75*
*p* **	*0.64*	*0.076*	*0.13*	
CD57^+^				
Overall CD8	34 (8.8–58)	45 (12–70)	36 (28–61)	*0.26*
HIV-specific	33 (15–85)	39 (20–73)	60 (53–71)	*0.20*
*p* **	*0.32*	*0.92*	*0.20*	
CD45RA^+^CD57^+^				
Overall CD8	14 (2.5–33)	15 (2.4–31)	32 (4.0–35)	*0.31*
HIV-specific	8.0 (3.0–57)	16 (6.7–42)	32 (12–70)	*0.21*
*p* **	*0.31*	*0.52*	*0.21*	
HLA-DR^+^				
Overall CD8	43 (35–62)	39 (24–51)	34 (32–35)	*0.15*
HIV-specific	81 (45–97)	79 (45–90)	80 (68–85)	*0.017*
*p* **	*0.017*	*0.0050*	*0.0032*	
CD95^+^				
Overall CD8	56 (8.4–78)	47 (16–63)	47 (34–61)	*0.33*
HIV-specific	92 (28–95)	83 (46–91)	88 (79–96)	*0.42*
*p* **	*0.027*	*0.010*	*0.030*	

Notes. Five infants have paired data at 3–6 and 7–12 month intervals, 3 infants have data at all 3 intervals.

*p value for comparison of 3–6 versus 23–24 month interval data.

**p value for comparison of overall CD8 versus HIV-specific (tetramer) data at each interval. No p values met significance following Holm Test for multiple comparisons.

Despite the small sample size and limited power, some longitudinal trends were observed. Notably, the frequency of CD27+CD28- cells predominated the HIV-specific CD8^+^ T cell subset throughout infection, and very few cells transitioned to lose CD27 expression by 23–24 months (mean 3.9%, range 1.1–6.1%). The proportion of cells expressing CD57 increased over time in the overall CD8^+^ T cell subset from 33% (range 15–85) at 3–6 months to 60% (range 53–71) at 23–24 months in the HIV-specific CD8^+^ T cell subset (p = 0.20). HLA-DR and CD95 expression remained high in both the overall CD8^+^ and HIV-specific CD8^+^ T cell subsets throughout infection.

In summary, both the overall and HIV-specific CD8^+^ T cell subsets underwent changes during early and chronic infection; the HIV-specific CD8^+^ T cell subset was remarkable in the high frequency of cells maintaining activation and pro-apoptotic markers during chronic infection.

## Discussion

In this study we determined the longitudinal frequency and phenotype of HIV-specific CD8^+^ T cells during primary and chronic infant HIV-1 infection. Infants were able to generate HIV-specific CD8^+^ T cells similar to adults in frequency and phenotype during early HIV-1 infection. However, during chronic infection, HIV-specific CD8^+^ T cells maintained an activated phenotype and expressed CD95, suggesting vulnerability to apoptosis. Persistent activation and expression of CD95 on HIV-specific CD8^+^ T cells may result in the apoptotic elimination of virus-specific T cells, further enabling the persistence of viral replication, and perhaps explaining the poor prognosis of HIV-1 infected infants. Alternatively, infant T cells may be compromised in their transition to a resting phenotype during early infection, and this in turn could facilitate viral replication.

During early HIV-1 infection in infants, we detected HIV-specific CD8^+^ T cells binding a single tetramer at frequencies as high as 3.9%, which is similar to those observed in adults (0.1–3%) [13], [28], [29], [30], and older children (0.1–2.4%) [19], [20], [31]. Though high frequencies of tetramer-positive cells were detected during infant HIV-1 infection, this did not confer a measurable advantage in the control of viral replication; infants maintained very high viral loads during the period of observation and two died during the study. Adult studies of acute HIV-1 infection have shown decreases in the frequency of tetramer-positive cells following the initiation of ART [30], [32]. In a single study of adults not on ART, tetramer-positive cells declined following acute infection, presumably due to effective host immune responses and concomitant reduction of HIV-1 load [33]. During chronic infection in adults, the frequency of tetramer-staining cells typically follows a pattern which closely follows changes in viral load [13]. In the 3 infants followed until 2 years of age, we observed a decline in tetramer-staining cells despite persistently high HIV-1 viral loads, suggesting high levels of antigen do not sustain continued HIV-specific CD8^+^ expansion.

The differentiation phenotype of infant HIV-specific CD8^+^ T cells was similar to adults; HIV-specific CD8^+^ T cells were primarily CD27^+^CD28^-^; a subset of infant CD8^+^ T cells expressed both CD45RA and CD57 and were activated, suggesting terminal differentiation to an effector phenotype (limited in proliferative potential but likely cytotoxic [34], [35]). This similarity in phenotype between infants and adults suggests that engagement of the infant T cell receptor results in propagation of a signal sufficient to drive cell division and differentiation to a similar stage as is reached in adults. It is also critical to note that although many of the changes we observed in the overall CD8^+^ T cell subset were likely due to the outgrowth of HIV-specific cells, the differentiation in the overall CD8^+^ T cell subset may also be influenced by co-infection with other viruses, particularly cytomegalovirus (CMV), which is known to drive the expansion of CD8^+^ cells with a CD27^−^CD28^−^, and CD57^+^ phenotype [36], [37]. Six of the seven children in our study had acquired CMV by 3 months of age (data not shown), suggesting that CMV infection may play an important role in shaping the characteristics of the infant overall CD8^+^ T cell subset.

In adults, the resolution of acute HIV-1 infection is accompanied by a transition of HIV-specific CD8^+^ T cells from a predominantly activated to a predominantly resting population following the reduction of viral load by therapy or host immune responses [32], [34]. In contrast, during our observation of 3 infants during the 2-year post infection period, infant HIV-specific CD8^+^ T cells remained predominantly activated, expressing high levels of HLA-DR and CD95. Activated cells are highly susceptible to activation induced cell death (AICD), and the expression of CD95 on HIV-specific CD8 confers additional susceptibility to Fas-mediated apoptosis [38]. HIV-infected macrophages and CD4^+^ T cells upregulate Fas-ligand (CD95L) expression on the cell surface, enabling them to initiate apoptosis in HIV-specific CTL expressing CD95 [39], [40]. Continuing activation and apoptosis may explain in part the reduction in the frequency of tetramer-staining cells over time. The persistent activation observed in infants may be a consequence of a failure to control HIV-1 replication during acute infection, but may also subsequently impair the immune response against HIV-1 during chronic infection. In addition to the direct effects of cellular activation on the survival and removal of HIV-specific cells, persistent cellular activation also has a more generalized negative effect on the host; cellular activation is a strong independent predictor of HIV-1 disease progression, and is likely to contribute significantly to the depletion of CD4^+^ T cells via yet undefined mechanisms [41], [42].

This study has several strengths and also some important weaknesses to note. Study strengths include the longitudinal assessment of individuals and the absence of confounding by antiretroviral therapy. By using cryopreserved specimens, we were able to batch all of each infant's specimens in a single experiment; greatly reducing intra-subject experimental error. A limitation of the study was that few infants in the larger cohort had ELISPOT responses of a magnitude and specificity to examine with available tetramers. These phenotypic data may therefore not be representative of HIV-specific CD8^+^ T cells directed at less dominant epitopes, or T cells from infants with low level responses. Additionally, the number of cellular antigens we were able to examine in the phenotypic studies were restricted by limited cell numbers in the cryopreserved specimens. Finally, the small sample size of this study limits our ability to perform meaningful statistical comparisons of the data; though some phenotypic markers differed between subsets and time intervals, adjustment for multiple comparisons retained no significant values.

In summary, our data suggest a model of infant infection in which high frequencies of phenotypically normal CD8^+^ T cells fail to contain viral replication during acute infection, resulting in persistent T cell activation during chronic infection. Identifying the mechanisms underlying age-related differential function of T cells will be valuable to the design of an HIV-1 vaccine appropriate for use in infants and to our general understanding of HIV-specific immunity.

## Materials and Methods

### Participants and cohort study design

The study was approved by the Ethics and Research Committee of Kenyatta National Hospital and the Institutional Review Board of the University of Washington. Mothers provided written informed consent for study participation on behalf of themselves and their infants. Details of the cohort recruitment and follow-up have been presented in detail elsewhere [9], [17], [43]. The cohort was enrolled and followed-up before antiretroviral therapy (ART) became widely available in Kenya; other than maternal antenatal zidovudine prophylaxis [44], infants did not receive ART. Serial blood specimens were obtained at delivery and months 1 and 3, and quarterly thereafter. Children were followed until death or exit from the study at two years of age. A subset of 7 infants was selected from this well-characterized cohort for immunologic studies based on timing of HIV-1 acquisition, previously characterized immune responses and availability of frozen specimens.

### HIV-1 diagnosis and viral loads

HIV-1 RNA viral loads were measured using the Gen-Probe transcription mediated assay [45]. Infant HIV-1 infection was diagnosed by nested PCR amplifying HIV-1 Gag proviral DNA from dried blood spotted onto filter paper [46]. Infant HIV-1 infection *in utero* was defined as the detection of either HIV-1 DNA or RNA within 48 hours of birth, followed by a positive specimen (viral RNA or DNA) at the subsequent clinic visit. Consistent with our other studies, the peak HIV-1 viral load was defined as the highest measurement obtained during the first 6 months of infection, and the set-point viral load was defined as the first viral load measured at least 6 weeks after the peak [47].

### IFN-γ ELISPOT Assay

Amplification refractory mutation system-PCR was used to determine infant HLA types [48]. Peptides were selected on the basis of HLA type from a panel of optimized HIV-1 subtype A and D CD8^+^ T cell epitopes previously identified in studies of Kenyan sex workers [17], [49]. Enzyme-linked immunospot assays (ELISPOT) were performed on freshly isolated PBMC obtained from EDTA anticoagulated blood as previously described [17], [50]. Spot forming units (SFU) was defined as the average number of spots in duplicate peptide test wells, and HIV-1-specific SFU (HIVSFU) was defined as SFU minus the average number of spots in the duplicate negative control wells.

### Class I HLA-tetramers and antibody and tetramer staining

The following tetramers were produced in our laboratory as previously described [28]: HLA-A03-Nef-QVPLRPMTYK, B08-Nef-FLKEKGGL, and B57-P24-KAFSPEVIPMF, A29-GP120-FNCGGEFFY. All tetramers were produced using extravidin-PE (Sigma, Gillingham, UK) as the fluorochrome label. HIV-1 infected infants who had acquired HIV-1 by 1 month of age, and had ELISPOT responses >400 HIVSFU/10^6^ PBMC in fresh assays were selected for tetramer staining. This threshold was chosen because tetramer staining is often unable to confirm ELISPOT responses below this level. All serial specimens from each individual infant were stained and analyzed on the same day to minimize intra-patient variation. Cryopreserved specimens were thawed in fetal calf serum (FCS) and stained with tetramer. Cells were stained with tetramer-PE, CD3-Pacific Blue (clone UCHT1, Dako Cytomation, Angel Drove, UK), CD8-APC-Cy7 (clone RPA-T8, Pharmingen), CD38-PE-Cy5 (clone HIT2, Pharmingen, Oxford, UK), CD28-FITC (clone CD28.1, Dako Cytomation), CD27-APC (clone 0323, eBioscience, San Diego, CA, USA), HLA-DR-PE-Cy5 (clone G45-6, Pharmingen), CD57-FITC (clone TB01, Serotec, Kidlington, UK) and CD45RA-APC (clone HI100, Pharmingen), and CD95-APC (clone DX2, Pharmingen). No phenotyping was available for infant B1-454, whose cells were stained with CD3, CD8 and tetramer only. Cells were acquired on a Cyan instrument using Summit software (Dako Cytomation) and analyzed with FlowJo software (Treestar, Inc., Olten Switzerland). HIV-1 negative donor cells from an HIV-uninfected adult were used to confirm an absence of staining for each tetramer. Gates for fluorochrome-conjugated antibodies were placed with the aid of isotype controls. Tetramer^+^ cells/million PBMC was calculated by dividing the number of events in the tetramer gate by the number of events acquired, excluding the cluster of FSC^verylow^/SSC^verylow^ events likely to be debris or necrotic cells. Further details of compensations, isotype controls, and gating schema can be found in File S1 accompanying this article.

### Statistical Analysis

Clinical and laboratory data were collected and stored in a Microsoft Access Database (Microsoft Inc., Redmond, Washington, USA) and analyzed using Stata SE v.11 for Macintosh (Stata Corp. College Station, Texas, USA). Viral loads were log_10_-tranformed before comparisons of means using the t-test. Median CD4 values were compared using the Mann Whitney U test. Comparisons of cellular phenotype between time intervals, and between the HIV-specific and overall CD8^+^ T cell subsets were performed using the paired t-test on log_10_-transformed data; one infant had >1 measurement within an interval, measurements were averaged for this individual. We used the Holm Test to evaluate p values with non-independent multiple comparisons [51]. All reported p values are two tailed.

## Supporting Information

File S1 (DOC)
