# Supplementary material for: Phenotypic Characterization of HIV-Specific CD8+ T Cells during Early and Chronic Infant HIV-1 Infection
Source: PLoS One. 2011 May 31;6(5):e20375. doi: 10.1371/journal.pone.0020375 (PMC3105047; doi:10.1371/journal.pone.0020375)
Supplement: File S1 — (DOC) [file pone.0020375.s001.doc]

**S1. Supporting information**

Compensation

Single stains were prepared for each fluorochrome using cryopreserved HIV-1 negative donor PBMC. The same donor cells were used for all experiments to enable monitoring of antibody and reagent quality. Compensation was performed manually before acquisition of cells; the CyAn performs digital compensation and stores the compensation matrix in the list mode file. For final analysis, we readjusted compensation using FlowJo’s automated compensation platform “Define New Matrix”.

Isotype controls

Isotype controls were also prepared for each run from the same donor control cells. We set the cutoff for each matched antibody to the 99th percentile.

Placement of gates

In order to minimize intra-patient variability and to enable comparison of relative fluorescence intensities, we thawed all cryopreserved specimens (stored from 1-7 time-points) from an individual infant on the same day for flow cytometry.

Gating overall CD8 and tetramer subsets:

1) Our first gate was made broadly to include large lymphocytes (to capture activated cells).

2) CD3+ cells were selected from the lymphocyte gate.

3) CD3+CD8+ double-positive cells were selected from the lymphocyte gate.

4) Tetramer-positive cells were selected from the CD3+ gate (to enable exclusion of non-specific staining in the CD8 negative subset).

5) The tetramer gate was pasted onto the CD3+CD8+ subset to obtain frequencies of tetramer+ cells as a proportion of CD3+CD8+ cells.

6) These gates were pasted across all time-points for an individual infant and checked, adjusted if necessary.

Algorithm for gating different phenotypic markers :

1) Apply cut-offs as indicated by isotype controls.

2) If these cut-offs do not appear appropriate (Eg, if they bisected what was clearly a distinct cluster), allow gates to move up in channel, but not down.

3) Once a gate placement was decided for an infant sample, gates were copied and pasted onto all additional sample time-points for that infant.
